# Feasibility of Shear Wave Elastography Imaging for Evaluating the Biological Behavior of Breast Cancer

**DOI:** 10.3389/fonc.2021.820102

**Published:** 2022-01-27

**Authors:** Chaoxu Liu, Jin Zhou, Cai Chang, Wenxiang Zhi

**Affiliations:** Department of Ultrasonography, Fudan University Shanghai Cancer Center, Shanghai Medical College, Fudan University, Shanghai, China

**Keywords:** ultrasound, shear wave elastography, breast cancer, prognosis, biological behavior

## Abstract

**Objective:**

To explore the feasibility of shear wave elastography (SWE) parameters for assessing the biological behavior of breast cancer.

**Materials and Methods:**

In this prospective study, 224 breast cancer lesions in 216 female patients were examined by B-mode ultrasound and shear wave elastography in sequence. The maximum size (S_max_) of the lesion was measured by B-mode ultrasound, and then shear wave elastography was performed on this section to obtain relevant parameters, including maximum elasticity (E_max_), mean elasticity (E_mean_), standard deviation of elasticity (SD), and the area ratio of shear wave elastography to B-mode ultrasound (AR). The relationship between SWE parameters and pathological type, histopathological classification, histological grade, lymphovascular invasion status (LVI), axillary lymph node status (ALN), and immunohistochemistry of breast cancer lesions was performed according to postoperative pathology.

**Results:**

In the univariate analysis, the pathological type and histopathological classification of breast cancer were not significantly associated with SWE parameters; with an increase in the histological grade of invasive ductal carcinoma (IDC), SD (*p* = 0.016) and S_max_ (*p* = 0.000) values increased. In the ALN-positive group, S_max_ (*p* = 0.004) was significantly greater than in the ALN-negative group; S_max_ (*p* = 0.003), E_max_ (*p* = 0.034), and SD (*p* = 0.045) were significantly higher in the LVI-positive group than in the LVI-negative group; SD (*p* = 0.043, *p* = 0.047) and S_max_ (*p* = 0.000, *p* = 0.000) were significantly lower in the ER^+^ and PR^+^ groups than in the ER^-^ and PR^-^ groups, respectively; AR (*p* = 0.032) was significantly higher in the ER^+^ groups than in the ER^-^ groups, and S_max_ (*p* = 0.002) of the HER2^+^ group showed higher values than that of the HER2^-^ group; S_max_ (*p* = 0.000), SD (*p* = 0.006), and E_max_ (*p* = 0.004) of the Ki-67 high-expression group showed significantly higher values than those of the Ki-67 low-expression group. In the multivariate analysis, Ki-67 was an independent factor of S_max_ (*p* = 0.005), E_max_ (*p* = 0.004), and SD (*p* = 0.006); ER was an independent influencing factor of S_max_ (*p* = 0.000) and AR (*p* = 0.032). LVI independently influences S_max_ (*p* = 0.006).

**Conclusions:**

The SWE parameters E_max_, SD, and AR can be used to evaluate the biological behavior of breast cancer.

## Introduction

Breast cancer is one of the most common cancers, and the mortality rate of breast cancer ranks first among women (1). Predicting the prognosis of breast cancer preoperatively can aid in understanding the disease course and guiding treatment. The pathological type, histopathological classification, histological grade, axillary lymph node status (ALN), lymphovascular invasion status (LVI), and immunohistochemical factors such as estrogen receptor (ER) and progesterone receptor (PR), human epidermal growth factor receptor 2 (HER2), and the Ki-67 proliferation index affect the prognosis of breast cancer (2–5), and these methods of evaluating the breast cancer prognosis are obtained invasively *via* biopsy. Therefore, noninvasive methods to assess the prognosis of breast cancer are urgently needed. Shear wave elastography (SWE) imaging is a recently emerging elastography technology, which can qualitatively and quantitatively differentiate between malignant and benign foci, and its clinical value has been widely recognized (6–9). As quantitative parameters of SWE, the maximum elasticity (E_max_), standard deviation of elasticity (SD), and mean elasticity (E_mean_) can quantitatively reflect the tissue hardness of the lesion, and the relationship between these parameters and patient clinicopathological characteristics has attracted considerable clinical attention (10). Determining whether these parameters can be used to quantitatively evaluate the biological behavior of breast cancer has been a major research focus in recent years (11, 12).

Kim’s research results showed that tumors with higher E_max_ and E_mean_ values were found to exhibit poor pathological differentiation (13). Huang et al. concluded that SD can reflect the heterogeneity of tumors and can be used to distinguish benign from malignant lesions. The SD of malignant tumors was found to be relatively high (14). Our research revealed that in addition to the higher E_max_ values of malignant foci, the elastic areas of some foci were larger than the B-mode areas. Does the hardness of the tissues surrounding the lesion change earlier than the morphology of the lesion? In Leong’s study, it was mentioned that the area ratio of shear wave elastography to B-mode ultrasound (AR) is helpful for identifying benign and malignant breast foci (15), but the clinicopathological characteristics of AR in different malignant foci were not discussed. In view of the fact that there are few studies in this area, we aim to explore the distribution characteristics and influencing factors of AR in different breast cancer types in our research. We also intend to explore the relationships between the SWE parameters and the clinicopathological characteristics of breast cancer patients to clarify the feasibility of using SWE parameters to predict the biological behavior of breast cancer preoperatively.

## Materials and Methods

### Patients

Our research obtained approval from the Shanghai Cancer Center Institutional Review Board, and all patients in this prospective study provided written informed consent. This work was conducted from January 2019 to December 2019 at Fudan University Shanghai Cancer Center (FUSCC). We prospectively recruited 354 breast patients with 362 breast foci for B-mode ultrasound (US) and SWE during this period. The maximum diameter of the collected breast lesions was less than or equal to 30 mm. Compared with postoperative and biopsy pathologies, 116 benign breast lesions were excluded. Another 22 breast patients were excluded for the reasons as follows: 7 lesions in 7 patients showed artifacts because of tumor prominence from the skin surface; 10 breast lesions in 10 patients were located beside or behind the nipple, which causes inaccuracy in elastography imaging; and 5 patients were lost to follow-up. Therefore, a total of 224 breast cancer foci from 216 patients were ultimately included.

### B-Mode Ultrasound and Shear Wave Elastography Examinations

B-mode US and SWE were performed by a Supersonic Aixplorer color Doppler ultrasound diagnostic apparatus (Supersonic Imagine, Aix en Provence, France) and which was equipped with an SL 15-4 MHz linear array transducer, and all imaging was collected by 2 sonographers with 5 and 10 years of working experience in breast US. In this study, the largest section of the lesion was selected to measure S_max_ in B-mode US examination. Then, the mode was switched to SWE for the largest section of the lesion to obtain the SWE image. The sampling frame was placed to contain the entire section of the lesion. The transducer remained relatively fixed for approximately 5 s without applying pressure to the lesion until the SWE image was stable and no artifacts were observed, and then the SWE image was obtained. Regions of interest (ROIs) were acquired respectively in shear wave elastography and B-mode ultrasound by drawing the outline of the border of the lesion to exactly include the lesion without breast tissue around the lesion. E_max_ value, E_mean_ value, and SD value of the entire lesion in the SWE image were generated automatically by the US system, as well as the area of the lesion in the B-mode US image and the area of the lesion in the SWE image, and then AR was calculated by dividing the area of SWE by the area of B-mode US. Each of the lesions was continuously obtained three times for three images, and then average values of three images were calculated to obtain the final results of the SWE parameters.

### Pathologic Analysis

The pathological evaluation parameters included ER and PR status, HER2 status, Ki-67 proliferation index, LVI status, ALN status, pathological type, histopathological classification, and histological grade. Histological grading was determined by the Nottingham grading system (16). The modified American Society of Clinical Oncology-College of American Physicians scoring guidelines were used (17, 18). Positive ER and PR status was ruled as ≥1% of tumor cells with positive nuclear staining. On the basis of standard criteria, the immunohistochemical staining of HER2 was scored as 0, 1+, 2+, and 3+. A score of 3+ was classified as HER2 positive, and a score of 0 and 1+ was classified as negative. There was another situation where tumors had a score of 2+, fluorescence *in situ* hybridization testing (FISH) was performed to identify the amplification of HER2, and a positive FISH amplification was defined to be HER2 positive. The cells with Ki-67 nuclear-stained were defined to be positively stained. Based on this criterion, the Ki-67 proliferation index was defined as the percentage of cells that were Ki-67-positive in greater than or equal to 500 tumor cells in the hot spot of each slide. High Ki-67 index expression was ruled as ≥14%, and low Ki-67 index expression was ruled as <14% (19). ALN positivity refers to axillary lymph node metastasis. LVI positivity was defined as the existence of cancer cells in the lymphatic and/or blood vessels in or around the tumor. Two senior physicians pathologically diagnosed the lesions, and a consensus was reached through discussion when disagreements occurred.

### Statistical Analysis

The Statistical Package for the Social Sciences for Windows (SPSS, Chicago, IL, USA, version 23.0) was used to perform all statistical analyses. A simple linear regression model was used to analyze the relationship between the SWE parameters and clinicopathological parameters. Stepwise multiple linear regression analysis was used to determine the pathological parameters independently related to the SWE parameters. A *p*-value < 0.05 was considered statistically significant.

## Results

### Clinical Characteristics

**Figure 1:**
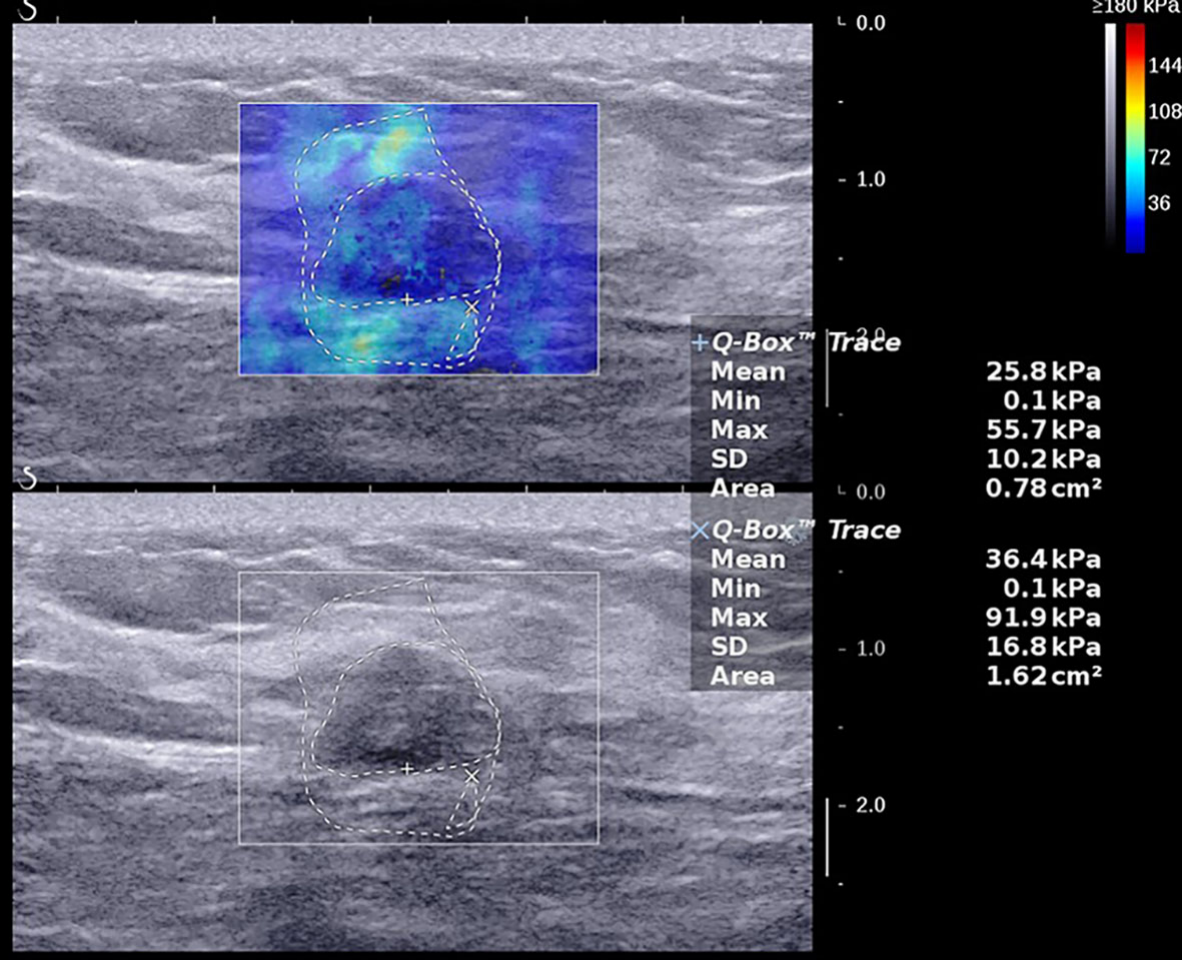
A 47-year-old female with a 14.0-mm lesion in the right breast. A freehand ROI was drawn manually to measure areas by drawing the outline of the border of the lesion respectively in shear wave elastography and B-mode ultrasound. The SWE values of the lesion were as follows: E_max_, 137.9 kPa; E_mean_, 44.4 kPa; SD, 27.2 kPa; AR, 2.29. The lesion was invasive ductal carcinoma with the histological grade II, showing negative lymphovascular invasion and negative axillary lymph node status, ER^+^ and PR^+^, HER2^-^, and low expression of Ki-67, 10%.

**Figure 2:**
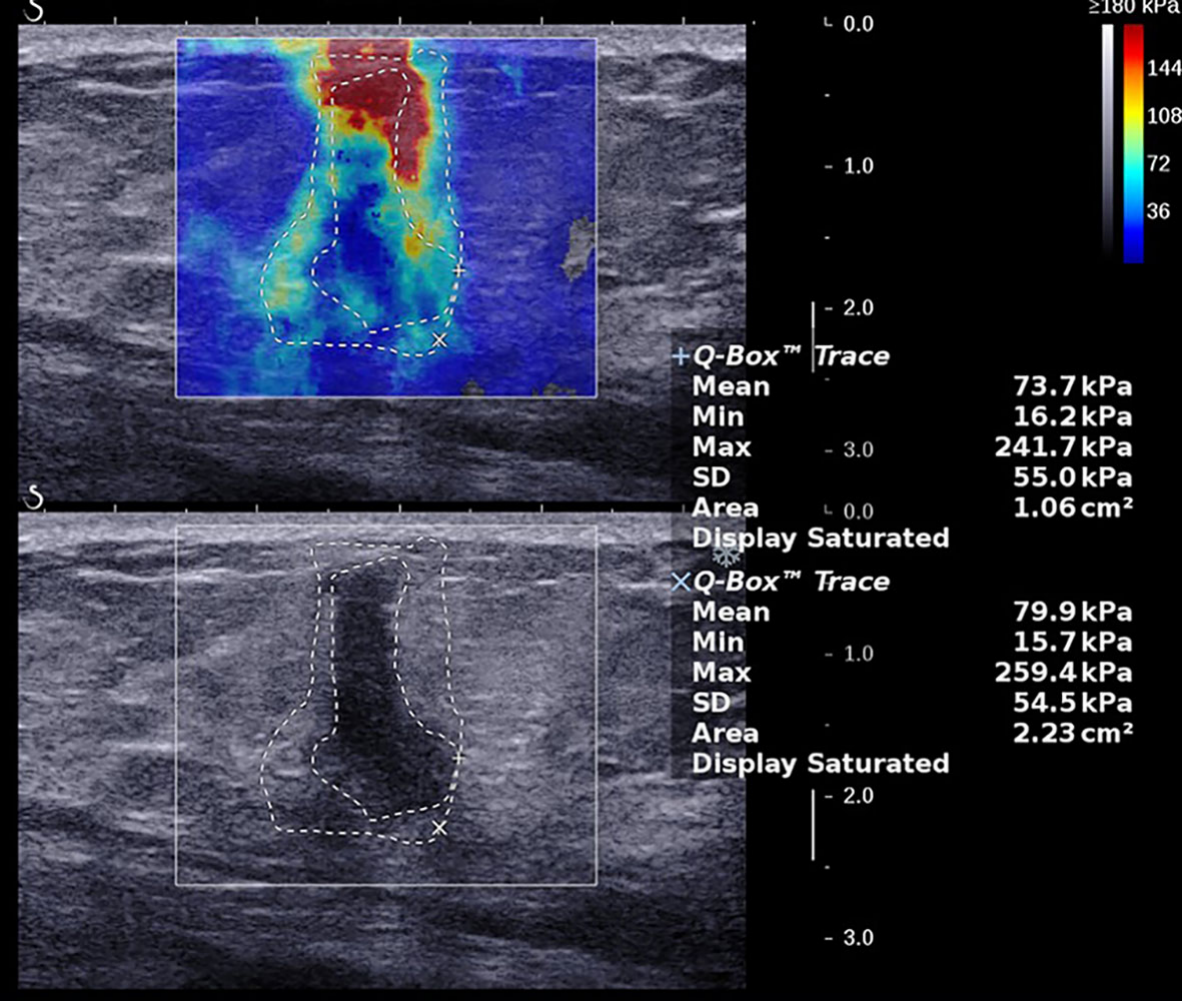
A 57-year-old female with a 15.0-mm lesion in the left breast. The SWE parameter values of the cancer were as follows: E_max_, 261.5 kPa; E_mean_, 82.4 kPa; SD, 54.2 kPa; AR, 1.95. The lesion was invasive ductal carcinoma with the histological grade II, showing negative lymphovascular invasion and negative axillary lymph node status, ER^-^ and PR^-^, HER2^+^, and high expression of Ki-67, 15%.

**Figure 3:**
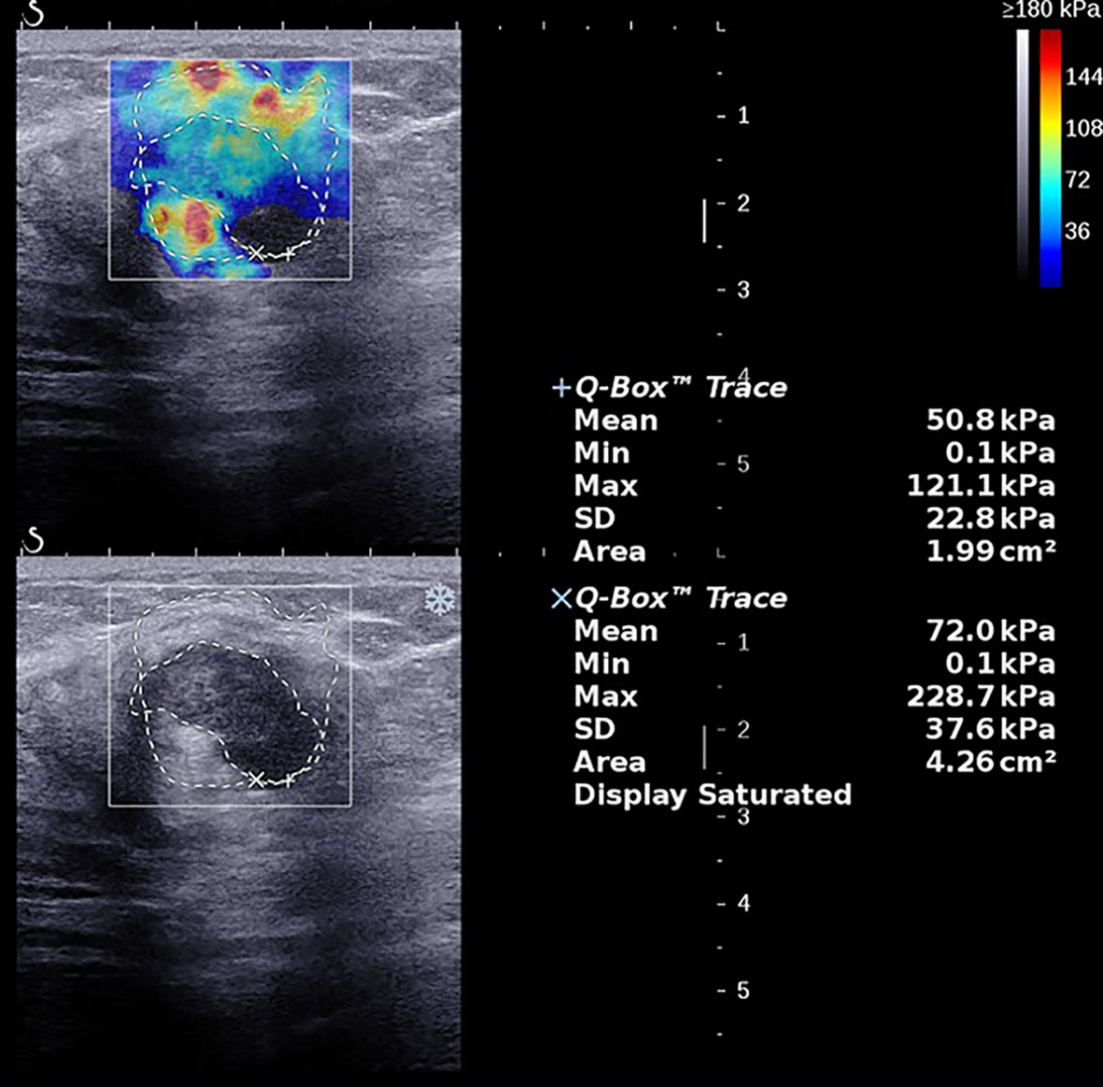
A 62-year-old female with a 28.0-mm lesion in the left breast. The SWE parameter values of the cancer were as follows: E_max_, 236.0 kPa; E_mean_, 69.3 kPa; SD, 41.5 kPa; AR, 2.02. The lesion was invasive ductal carcinoma with the histological grade III, showing negative lymphovascular invasion and negative axillary lymph node status, ER^-^ and PR^-^, HER2^-^, and high expression of Ki-67, 60%.

**Figure 4:**
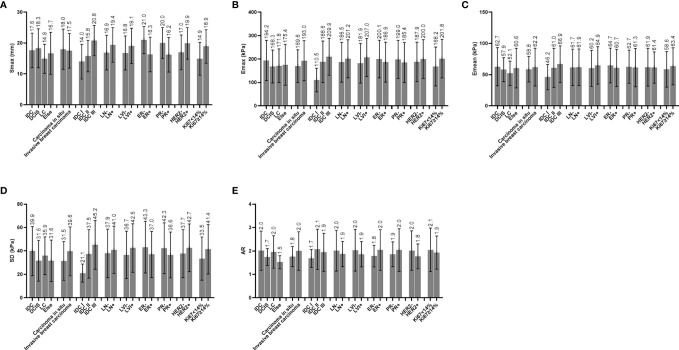
**(A)** Distribution characteristics of S_max_ between different groups. **(B)** Distribution characteristics of E_max_ between different groups. **(C)** Distribution characteristics of E_mean_ between different groups. **(D)** Distribution characteristics of SD between different groups. **(E)** Distribution characteristics of AR between different groups; IDC, invasive ductal carcinoma; DCIS, ductal carcinoma in situ; LC, lobular carcinoma; ALN, axillary lymph node status; LVI, lymphovascular invasion; ER, estrogen receptor; PR, progesterone receptor; HER2, human epidermal growth factor receptor 2; Ki67, Ki67 index; S_max_, maximal size; E_max_, maximal elasticity; E_mean_, mean elasticity; SD, standard deviation of elasticity; AR, area ratio of shear wave elastography to B-mode ultrasound.

The average age of the 216 breast cancer patients was 53.7 ± 10.6 years (range 28–79 years), the average size of the 224 foci was 17.6 ± 5.7 mm (range 5.0–30.0 mm), and the average values of the SWE parameters were as follows: E_max_ 190.4 ± 84.1 kPa (range 12.0–300.0 kPa), E_mean_ 61.8 ± 29.3 kPa (range 4.0–169.5 kPa), SD 38.7 ± 20.5 kPa (range 2.6–87.9 kPa), and AR 1.97 ± 0.79 (range 0.82–8.05), and the AR values of all lesions were higher than 1 except for one lesion, which was 0.82 (**Figures 1**–**3**). The B-mode US and SWE parameters of the lesions are listed in (**Figure 4**).

A total of 224 breast cancer foci were observed among 216 breast cancer patients. The pathological types of cancers included 188 lesions of invasive ductal carcinomas (IDC), 21 lesions of ductal carcinomas *in situ* (DCIS), 7 lesions of invasive lobular breast carcinomas (ILC), 1 lesion of lobular carcinoma *in situ*, 3 lesions of intraductal papillary carcinomas, 1 lesion of solid intraductal papillary carcinoma with interstitial infiltration, 2 lesions of mucinous adenocarcinomas, and 1 lesion of invasive carcinoma with mucus secretion.

### Simple Linear Regression Analysis of the Correlation between SWE Parameters and Clinicopathological Parameters

#### Pathological Type, Histopathological Classification, and Histological Grade

In this study, pathological type had no correlation with SWE parameters (**Table 1**). On histopathological classification, there were 199 cases of invasive breast cancer and 25 cases of cancer *in situ*. The E_max_ (*p* = 0.190) and SD (*p* = 0.062) values of the invasive breast cancer group were higher than those of the cancer *in situ* group, but no significant differences were observed (**Table 1**).

**Table 1:** Univariate analysis of correlation of pathological type and histopathological classification with B-mode ultrasound and shear wave elastography parameters.

Variable		S_max_		E_max_		E_mean_		SD		AR	
	*n*	*p*	*β* (95% CI)	*p*	*β* (95% CI)	*p*	*β* (95% CI)	*p*	*β* (95% CI)	*p*	*β* (95% CI)
Pathological type											
IDC	188	0.583	−0.716 (−3.287 to 1.854)	0.182	25.933 (−12.246 to 64.113)	0.484	4.739 (−8.577 to 18.055)	0.080	8.280 (−1.009 to 17.569)	0.128	0.278 (−0.080 to 0.636)
DCIS	21	\		\		\		\		\	
LC	8	0.143	−3.458 (−8.100 to 1.183)	0.919	3.544 (−65.400 to 72.488)	0.634	−5.826 (−29.871 to 18.220)	0.611	4.330 (−12.444 to 21.103)	0.500	0.222 (−0.425 to 0.869)
Else	7	0.514	−1.619 (−6.495 to 3.257)	0.846	7.148 (−65.274 to 79.570)	0.834	2.690 (−22.568 to 27.949)	0.999	−0.010 (−17.629 to 17.610)	0.535	−0.214 (−0.894 to 0.465)
Histopathological classification											
cancer *in situ*	25	\		\		\		\		\	
invasive breast cancer	199	0.707	−0.542 (−2.824 to 1.919)	0.190	23.432 (−11.676 to 58.540)	0.589	3.363 (−8.893 to 15.619)	0.062	8.121 (−0.419 to 16.661)	0.161	0.236 (−0.095 to 0.567)

IDC, invasive ductal carcinoma; DCIS, ductal carcinoma in situ; LC, lobular carcinoma; S_max_, maximal size; E_max_, maximal elasticity; E_mean_, mean elasticity; SD, standard deviation of elasticity; AR, area ratio of shear wave elastography to B-mode ultrasound.

p < 0.05 was considered statistically significant.

Since most of the breast cancer cases in this research were IDC and there were fewer other pathological types of breast cancers, IDC cases were chosen to be analyzed for the relationship between histological grade and SWE parameters. In this study, there were 188 cases of IDC (including 1 case with missing histological grading information), including 4 cases of IDC-I, 115 cases of IDC-II, and 68 cases of IDC-III. Although the E_max_ (*p* = 0.072), E_mean_ (*p* = 0.339), and SD (*p* = 0.119) values were not significantly different between IDC-I and IDC-II, the average values of these parameters in the IDC-II group was greater than those in the IDC-I group. S_max_ (*p* = 0.000) and SD (*p* = 0.016) of the IDC-III group showed significantly greater values than those of the IDC-II group. The S_max_ (*p* = 0.010), E_max_ (*p* = 0.024), and SD (*p* = 0.024) in the group of IDC-III showed significantly higher values than those in the group of IDC-I (**Table 2**).

**Table 2:** Univariate analysis of correlation of histological grading with B-mode ultrasound and shear wave elastography parameters.

Variable		S_max_		E_max_		E_mean_		SD		AR	
	*n*	*p*	*β* (95% CI)	*p*	*β* (95% CI)	*p*	*β* (95% CI)	*p*	*β* (95% CI)	*p*	*β* (95% CI)
Histological											
grading of IDC											
I	4	0.010	−6.809 (−11.945 to −1.673)	0.024	−99.363 (−185.467 to −13.260)	0.189	−20.634 (−51.483 to 10.215)	0.024	−24.090 (−45.032 to −3.147)	0.555	−0.255 (−1.107 to 0.597)
II	115	0.000	−4.983 (−6.510 to −3.456)	0.103	−21.281 (−46.882 to 4.320)	0.212	−5.820 (−14.992 to 3.353)	0.016	−7.662 (−13.889 to −1.435)	0.277	0.140 (−0.113 to 0.393)
III	68	\		\		\		\		\	

IDC, invasive ductal carcinoma; S_max_, maximal size; E_max_, maximal elasticity; E_mean_, mean elasticity; SD, standard deviation of elasticity; AR, area ratio of shear wave elastography to B-mode ultrasound.

p < 0.05 was considered statistically significant.

#### Axillary Lymph Node Status and Lymphovascular Invasion Status

There were 60 lesions in the ALN-positive group and 164 lesions in the ALN-negative group, and there were 76 lesions in the LVI-positive group and 148 lesions in the LVI-negative group. The S_max_ (*p* = 0.004) of the lesions showed lower values in the group of ALN-negative than that in the group of ALN-positive. E_max_ (*p* = 0.034), SD (*p* = 0.045), and S_max_ (*p* = 0.003) in the LVI-positive group were significantly higher than those in the LVI-negative group. Although the distributions were not significantly different, AR in the ALN-positive group (*p* = 0.237) was lower than in the ALN-negative group and AR in the LVI-positive group (*p* = 0.143) was lower than in the LVI-negative group (**Table 3**).

**Table 3:** Univariate analysis of correlation of ALN, LVI, and immunohistochemical biomarkers with B-mode ultrasound and shear wave elastography parameters.

	Variable	*N*	S_max_		E_max_		E_mean_		SD		AR	
			*p*	*β* (95% CI)	*p*	*β* (95% CI)	*p*	*β* (95% CI)	*p*	*β* (95% CI)	*p*	*β* (95% CI)
ALN	ALN^-^	164	\		\		\		\		\	
	ALN^+^	60	0.004	2.448 (0.792 to 4.103)	0.248	14.699 (−10.286 to −10.286)	0.970	0.169 (−8.551 to 8.889)	0.318	3.102 (−3.004 to 9.209)	0.237	−0.142 (−0.377 to 0.094)
LVI	LVI^-^	148	\		\		\		\		\	
	LVI^+^	76	0.003	2.342 (0.795 to 3.889)	0.034	25.086 (1.882 to 48.289)	0.252	4.735 (−3.397 to 12.867)	0.045	5.807 (0.135 to 11.480)	0.143	−0.164 (−0.384 to 0.056)
ER	ER^-^	59	\		\		\		\		\	
	ER^+^	165	0.000	−4.719 (−6.296 to −3.142)	0.301	−13.229 (−38.363 to 11.906)	0.373	−3.967 (−12.718 to 4.784)	0.043	−6.295 (−12.391 to −0.198)	0.032	0.257 (0.022 to 0.492)
PR	PR^-^	82	\		\		\		\		\	
	PR^+^	142	0.000	−3.794 (−5.262 to −2.327)	0.245	−13.574 (−36.541 to 9.394)	0.720	−1.458 (−9.472 to 6.556)	0.047	−5.646 (−11.223 to −0.070)	0.106	0.178 (−0.038 to 0.394)
HER2	HER2^-^	178	\		\		\		\		\	
	HER2^+^	46	0.002	2.936 (1.128 to 4.744)	0.384	12.140 (−15.285 to 39.566)	0.927	−0.443 (−10.003 to 9.116)	0.141	5.003 (−1.674 to 11.679)	0.055	−0.251 (−0.508 to 0.006)
Ki67	<14%	76	\		\		\		\		\	
	≥14%	148	0.000	3.971 (2.483 to 5.459)	0.004	33.541 (10.525 to 56.557)	0.247	4.785 (−3.347 to 12.916)	0.006	7.900 (2.272 to 13.528)	0.259	−0.127 (−0.347 to 0.094)

ALN, axillary lymph node status; LVI, lymphovascular invasion; ER, estrogen receptor; PR, progesterone receptor; HER2, human epidermal growth factor receptor 2; Ki67, Ki67 index; S_max_, maximal size; E_max_, maximal elasticity; E_mean_, mean elasticity; SD, standard deviation of elasticity; AR, area ratio of shear wave elastography to B-mode ultrasound.

p < 0.05 was considered statistically significant.

#### Immunohistochemical Factors

SD and S_max_ were significantly correlated with ER and PR status. Compared with the ER^-^ and the PR^-^ lesions, the ER^+^ and the PR^+^ lesions had lower SD (*p* = 0.043, *p* = 0.047) and S_max_ (*p* = 0.000, *p* = 0.000) values. AR (*p* = 0.032) was significantly higher in the ER^+^ lesions than in the ER^-^ lesions. AR (*p* = 0.106) in the PR^+^ lesions was also higher than in the PR^-^ lesions but there were no significant differences. The S_max_ (*p* = 0.002) of the HER2^+^ foci had significantly higher values than that of the HER2^-^ foci. The E_max_ (*p* = 0.004), SD (*p* = 0.006), and S_max_ (*p* = 0.000) values of the lesions in the Ki-67 high-expression group were significantly higher than those in the Ki-67 low-expression group. The AR (*p* = 0.055) of the HER2^+^ lesions was lower than those of the HER2^-^ lesions, and AR (*p* = 0.259) in the Ki-67 high-expression group was also lower than that in the Ki-67 low-expression group, but both no significant differences were observed (**Table 3**).

### Multivariate Analysis

It was shown in the multiple linear regression analysis that the Ki-67 index was an independent influencing factor of E_max_ (*p* = 0.004), SD (*p* = 0.006), and S_max_ (*p* = 0.005); ER status and LVI were independent influencing factors of Smax (*p* = 0.000, *p* = 0.006), and ER status independently influenced AR (*p* = 0.032) (**Table 4**).

**Table 4:** Multivariate linear regression analysis for clinicopathological factors.

	Variable	Smax		Emax		SD		AR	
		*p*	*β* (95% CI)	*p*	*β* (95% CI)	*p*	*β* (95% CI)	*p*	*β* (95% CI)
LVI	LVI^-^	\							
	LVI^+^	0.006	2.208 (0.586 to 3.469)						
ER	ER^-^	\						\	
	ER^+^	0.000	−3.838 (−5.491 to −2.184)					0.032	0.257 (0.022 to 0.492)
Ki67	<14%	\		\		\			
	≥14%	0.005	2.239 (0.677 to 3.801)	0.004	33.541 (10.525 to 56.557)	0.006	7.900 (2.272 to 13.528)		

LVI, lymphovascular invasion; ER, estrogen receptor; Ki67, Ki67 index; S_max_, maximal size; E_max_, maximal elasticity; SD, standard deviation of elasticity; AR, area ratio of shear wave elastography to B-mode ultrasound.

p < 0.05 was considered statistically significant.

## Discussion

This study explored the relationship between SWE parameters and clinicopathological characteristics. The results showed that in the univariate analysis, there was no significant difference in the distribution of SWE parameters among different pathological types of breast cancers, which is consistent with the conclusions reported by Ganau et al. (20), who indicated that pathological type is not the main factor affecting SWE parameters. Invasive breast cancer and cancer *in situ* have different biological behaviors and different clinical treatment plans. Therefore, we divided breast cancers into invasive breast cancer group and cancer *in situ* group. Compared with the cancers *in situ*, the invasive breast cancers showed higher values of E_max_ (*p* = 0.190) and SD (*p* = 0.062). As the aggressiveness of the lesion increases, both E_max_ and SD may increase.

The histological grade of IDC affects the clinical characteristics of the tissue. Our research clarified that lesions become harder and more heterogeneous with an increasing histological grade. The SD (*p* = 0.016) and S_max_ (*p* = 0.000) in the group of IDC-III were significantly higher than those in the group of IDC-II, and the S_max_ (*p* = 0.010), E_max_ (*p* = 0.024), and SD (*p* = 0.024) in the group of IDC-III showed higher values than those in the group of IDC-I. Even though the E_max_ (*p* = 0.072), E_mean_ (*p* = 0.339), and SD (*p* = 0.119) in the group of IDC-II were greater than those in the group of IDC-I, the SWE parameter distribution between the two groups did not show significant differences. This may be due to the small number of cases in the IDC-I group. The study by Evans et al. obtained similar results, showing that a higher histological grade was positively associated with a higher E_mean_ (21).

Our results showed that in the ALN-positive group, S_max_ (*p* = 0.004) showed greater values than that in the ALN-negative group, indicating that larger masses have a higher risk of ALN metastasis. There were no significant differences in the distribution of SWE parameters between the ALN-positive group and the ALN-negative group; E_mean_ (*p* = 0.970) values in the ALN-positive group and the ALN-negative group were similar; however, E_max_ (*p* = 0.248) in the ALN-positive group was greater than that in the ALN-negative group. The result is consistent with the report by Xue et al., in which they concluded that the E_max_ values in the breast cancer ALN-negative group were lower than that in the ALN-positive group (*p* = 0.110) but without significant differences (22). ALN can reflect prognosis, and it has been reported that E_mean_ or E_max_ of breast cancer in the ALN-positive group has higher Young’s modulus value (23, 24).

LVI positivity means that tumor cells have infiltrated the lymphatic and/or blood vessels in the area around the tumor, which is one of the critical steps of metastasis and is related to a poor prognosis (25, 26). The research by Son et al. indicated that the state of LVI is associated with E_mean_ and E_max_, with both values being higher in the LVI-positive group (27). In our research, the E_max_ (*p* = 0.034), SD (*p* = 0.045), and S_max_ (*p* = 0.003) of the LVI-positive group showed significantly higher values than those of the LVI-negative group, indicating that larger lesions become harder and more heterogeneous and are more likely to be LVI-positive. Therefore, preoperative E_max_ and SD values can infer whether a lesion has progressed to be LVI-positive. However, our research did not find a correlation between LVI and E_mean_. In the multiple linear regression analysis, the LVI did not show an independent correlation with E_max_ and SD, which may be due to the correlation between those two parameters and Ki-67. LVI-positive is often correlated with a high Ki-67 index (28).

ER, PR, HER2, and Ki-67 are markers used to distinguish breast cancer subtypes, and their expression status affects patients’ clinical treatment plan and prognosis (29). Our research results showed that the SD (*p* = 0.043, *p* = 0.047) and S_max_ (*p* = 0.000, *p* = 0.000) values of the ER^+^ and the PR^+^ lesions were smaller than those of the ER^-^ and the PR^-^ lesions. AR (*p* = 0.032) was significant higher in the ER^+^ lesions than in the ER^-^ lesions. We also found that AR (*p* = 0.106) was higher in the PR^+^ lesions, but there were no significant differences. Existing research results have indicated that ER^-^ and PR^-^ breast cancers tend to a worse prognosis than ER^+^ or PR^+^ breast cancers, while ER^+^ and PR^+^ tumors are well differentiated and less aggressive to a large extent (30). Therefore, based on the patient’s preoperative S_max_, SD, and AR values, we can infer the expression status of ER and PR. ER status independently affected S_max_ (*p* = 0.000) and AR (*p* = 0.032) in the multiple linear regression analysis, but failed to show an independent association with SD. This can be attributed to the relationship between SD and Ki-67, as ER^-^ tumors tend to have a higher Ki-67 index (31). In addition, our research shows that HER2 status is correlated with S_max_ (*p* = 0.002). The S_max_ value in the HER2^+^ group was larger than that in the HER2^-^ group, which may be a result from the high degree of malignancy and faster growth rate of the HER2^+^ lesions (32). Our study also found that the E_max_ and SD values of the HER2^+^ lesions were higher than those of the HER2^-^ lesions, but no significant differences were observed. At present, all the evidence from clinical studies have indicated that ER^+^, PR^+^, and HER2^-^ tumors have a good prognosis and show an excellent response to hormone therapy (33–37). This conclusion coincides with our research results, which show that ER^+^, PR^+^, and HER2^-^ tumors have smaller sizes, lower E_max_ and SD values, and higher AR values in most cases, which indicates a better prognosis.

Ki-67 is a nuclear antigen that reflects the proliferation activity of tumor cells, and its expression level is related to the degree of tumor malignancy. The higher the Ki-67 index, the more rapid the rate of tumor proliferation is, and the stronger the invasion and metastasis abilities are (38–40). In the univariate analysis, the E_max_ (*p* = 0.004), SD (*p* = 0.006) and S_max_ (*p* = 0.000) values of the lesions in the Ki-67 high-expression group showed significantly higher values than those in the Ki-67 low-expression group. In the multiple regression analysis, the Ki-67 index was also an independent influencing factor of E_max_ (*p* = 0.007), SD (*p* = 0.007), and S_max_ (*p* = 0.006). In this study, the higher SD values in the Ki-67 high-expression group may be triggered by the following mechanism: the more vigorous the cell karyokinesis is during tumor growth, the higher the Ki-67 index will be, which, in turn, increases the SD value, reflecting the heterogeneity in tumor cells. Therefore, the SD value can be used to noninvasively assess the biological behavior of cancer cells *in vivo*. E_max_ represents the hardness of the lesion, which is affected by the structure of necrotic tissue and fibrous tissue. In this study, tumors with a higher Ki-67 value had a larger E_max_ value, which is similar to the results of Nishimura et al. (41). This may explain why ischemic and hypoxic necrosis occurs inside lesions with high rates of tumor proliferation, which subsequently leads to fibrosis and hyperplasia, thereby increasing the hardness of the tumor (42, 43). In the Ki-67 high-expression group, AR (*p* = 0.259) was lower than in the Ki-67 low-expression group, although no significant differences were observed. In B-mode US images, infiltrating growth of the tumor does not change the acoustic impedance, but the shear wave elastic velocity of the tumor is changed, so the shear wave elastic area of the tumor could be larger than the B-mode image area, which may explain why the AR value is higher in malignant tumors than in benign breast foci. The AR values of all lesions in our study were higher than 1 except for one lesion, which was 0.82. We speculated that the worse the prognosis of the tumor, the more likely visible the surrounding infiltrated parts of the tumor on B-mode US, then the area of B-mode US will be larger, but the area of SWE may not increase proportionally, so AR values may be lower. On the contrary, the better the prognosis of breast cancer, the higher its AR value. So, this may explain why AR was higher in ALN-negative, LVI-negative, ER^+^, PR^+^, HER2^-^, and Ki-67 low-expression groups, which means better prognosis in our research.

This study has some shortcomings that should be noted: the number of pathological types of breast cancer cases showed a large difference in their distribution, with IDC being the majority of cases, which is related to the different incidences of different breast cancer types. We hope to expand the sample size in the future to further explore the distribution of SWE parameters in different pathological types of breast cancers.

## Conclusion

The shear wave elastography parameters E_max_, SD, and AR can be used to evaluate the biological behavior of breast cancer.

## Data Availability Statement

The original contributions presented in the study are included in the article/supplementary material. Further inquiries can be directed to the corresponding authors.

## Ethics Statement

The studies involving human participants were reviewed and approved by the Fudan University Shanghai Cancer Center Institutional Review Board. The patients/participants provided their written informed consent to participate in this study.

## Author Contributions

CL, JZ, and WZ offered the conception and design of the study. CC offered administrative support. CL and WZ collected the clinical and image data. CL and JZ conducted data analysis and interpretation. CL and JZ wrote the manuscript. WZ and CC reviewed and re-edited the manuscript. All authors contributed to the article and approved the submitted version.

## Funding

This study was supported by the National Natural Science Foundation of China (81801701 and 81830058).

## Conflict of Interest

The authors declare that the research was conducted in the absence of any commercial or financial relationships that could be construed as a potential conflict of interest.

## Publisher’s Note

All claims expressed in this article are solely those of the authors and do not necessarily represent those of their affiliated organizations, or those of the publisher, the editors and the reviewers. Any product that may be evaluated in this article, or claim that may be made by its manufacturer, is not guaranteed or endorsed by the publisher.
